# Tolvaptan for Children and Adolescents with Autosomal Dominant Polycystic Kidney Disease: Randomized Controlled Trial

**DOI:** 10.2215/CJN.0000000000000022

**Published:** 2022-01-18

**Authors:** Djalila Mekahli, Lisa M. Guay-Woodford, Melissa A. Cadnapaphornchai, Larry A. Greenbaum, Mieczyslaw Litwin, Tomas Seeman, Ann Dandurand, Lily Shi, Kimberly Sikes, Susan E. Shoaf, Franz Schaefer

**Affiliations:** 1 PKD Research Group, Department of Cellular and Molecular Medicine, KU Leuven, Leuven, Belgium; 2 Department of Pediatric Nephrology, University Hospital of Leuven, Leuven, Belgium; 3 Center for Translational Research, Children's National Research Institute, Washington, DC; 4 Rocky Mountain Pediatric Kidney Center, Rocky Mountain Hospital for Children at Presbyterian/St. Luke's Medical Center, Denver, Colorado; 5 Division of Pediatric Nephrology, Department of Pediatrics, Emory University School of Medicine and Children's Healthcare of Atlanta, Atlanta, Georgia; 6 Department of Nephrology, Kidney Transplantation and Arterial Hypertension, Children's Memorial Health Institute, Warsaw, Poland; 7 Department of Pediatrics, 2nd Faculty of Medicine, Charles University and Motol University Hospital, Prague, Czech Republic; 8 Department of Pediatrics, Dr. von Hauner Children's Hospital, LMU Munich, Munich, Germany; 9 Cerevel Therapeutics, Cambridge, Massachusetts; 10 Otsuka Pharmaceutical Development & Commercialization, Princeton, New Jersey (former); 11 Otsuka Pharmaceutical Development & Commercialization, Rockville, Maryland; 12 Otsuka Pharmaceutical Development & Commercialization, Princeton, New Jersey; 13 Division of Pediatric Nephrology, University Children's Hospital Heidelberg, Heidelberg, Germany

## Abstract

**Background:**

Tolvaptan slows expansion of kidney volume and kidney function decline in adults with autosomal dominant polycystic kidney disease (ADPKD). Progression during childhood could be treated before irreversible kidney damage occurs, but trial data are lacking. We evaluated the safety and efficacy of tolvaptan in children/adolescents with ADPKD.

**Methods:**

This was the 1-year, randomized, double-blind, portion of a phase 3b, two-part trial being conducted at 20 academic pediatric nephrology centers. Key eligibility criteria were ADPKD and eGFR ≥60 ml/min per 1.73 m^2^. Participants aged 12–17 years were the target group (group 1, enrollment goal *n*≥60); participants aged 4–11 years could additionally enroll (group 2, anticipated enrollment approximately 40). Treatments were tolvaptan or placebo titrated by body weight and tolerability. Coprimary end points, change from baseline in spot urine osmolality and specific gravity at week 1, assessed inhibition of antidiuretic hormone activity. The key secondary end point was change in height-adjusted total kidney volume (htTKV) to month 12 in group 1. Additional end points were safety/tolerability and quality of life. Statistical comparisons were exploratory and *post hoc*.

**Results:**

Among the 91 randomized (group 1, *n*=66; group 2, *n*=25), least squares (LS) mean reduction (±SEM) in spot urine osmolality at week 1 was greater with tolvaptan (−390 [28] mOsm/kg) than placebo (−90 [29] mOsm/kg; *P*<0.001), as was LS mean reduction in specific gravity (−0.009 [0.001] versus −0.002 [0.001]; *P*<0.001). In group 1, the 12-month htTKV increase was 2.6% with tolvaptan and 5.8% with placebo (*P*>0.05). For tolvaptan and placebo, respectively, 65% and 16% of subjects experienced aquaretic adverse events, and 2% and 0% experienced hypernatremia. There were no elevated transaminases or drug-induced liver injuries. Four participants discontinued tolvaptan, and three discontinued placebo. Quality-of-life assessments remained stable.

**Conclusions:**

Tolvaptan exhibited pharmacodynamic activity in pediatric ADPKD. Aquaretic effects were manageable, with few discontinuations.

**Clinical Trial registry name and registration number:**

Safety, Pharmacokinetics, Tolerability and Efficacy of Tolvaptan in Children and Adolescents With ADPKD (Autosomal Dominant Polycystic Kidney Disease) NCT02964273.

## Visual Abstract

**Figure:**
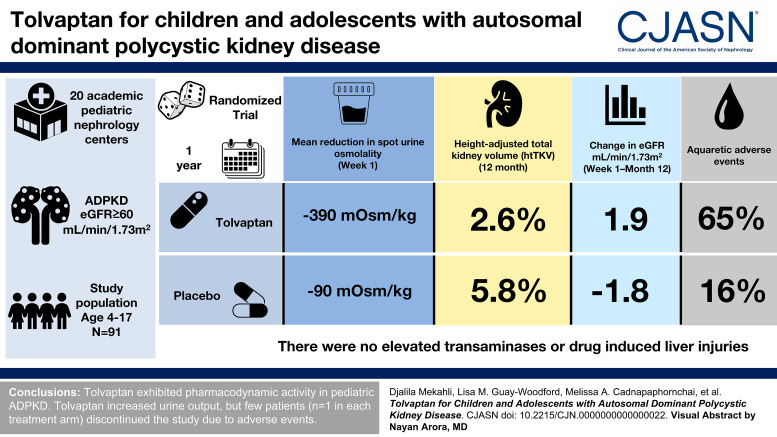

## Introduction

Autosomal dominant polycystic kidney disease (ADPKD) is one of the most common hereditary kidney disorders, characterized by the progressive formation and growth of cysts, leading to impairment of kidney function and eventually kidney failure.^1^ Cyst formation often initiates *in utero*, with heterogeneity in disease progression rates.^1–3^ In childhood, some patients exhibit early disease manifestations such as hypertension, decreased urinary concentrating capacity, and proteinuria,^1,4–9^ but relatively few exhibit overt symptoms common in adults, such as pain, hematuria, and urinary tract or cyst infections, despite progression of structural kidney disease.^2^

Given the phenotypic variability, uncertainty exists regarding the management of children with ADPKD or those at risk because of family history.^10^ Small cohort studies have been conducted, but in the absence of data from large-scale and/or randomized trials, questions about disease course and treatment remain unanswered, especially for children at increased risk of rapid progression on the basis of such factors as the presence of proteinuria, greater kidney volume, or rapid kidney volume growth.^1,2,11^

The management of ADPKD in adults has been changed by the vasopressin V2 receptor antagonist tolvaptan. In the TEMPO 3:4 clinical trial, tolvaptan inhibited the cyst-driven increase in total kidney volume (TKV) and slowed the decline in kidney function in adults at risk of rapid disease progression, with effects on kidney function confirmed in the REPRISE trial.^12,13^ Initiating tolvaptan in children at risk of rapidly progressing disease, before the kidney has been irreversibly damaged by cyst growth, could potentially achieve greater long-term benefits in delaying loss of kidney function than is possible in adults. An analysis of data on TEMPO 3:4 participants aged 18–24 years (*n*=51) supported therapeutic benefit in adolescents and young adults.^14^ However, pediatric trials of tolvaptan in ADPKD are lacking, and there are no approved therapies for pediatric ADPKD.^2,15,16^ Moreover, scoring systems to stratify patients into risk groups exist for adults with ADPKD, but not for children.^1,17–20^ Clinical trial data on tolvaptan in children and adolescents with ADPKD, in conjunction with efforts to identify determinants of progression, have the potential to improve long-term outcomes.

We conducted a phase 3, 1-year, randomized, double-blind, placebo-controlled, multicenter trial in participants with ADPKD aged 4–17 years. The trial objectives were to evaluate the pharmacodynamics, safety, and efficacy of tolvaptan in children and adolescents with ADPKD.

## Methods

### Participants

Study methods have been reported.^21^ Participants were enrolled at 20 academic pediatric nephrology centers in four countries (Belgium, Germany, Italy, and the United Kingdom) from September 2016 to November 2018. Key inclusion criteria were ADPKD diagnosis, eGFR by bedside Schwartz formula ≥60 ml/min per 1.73 m^2^, and body weight ≥20 kg.^22^ The target population was aged 12–17 years (group 1). Participants aged 4–11 years meeting eligibility criteria (group 2) were also allowed to enter during the recruitment of the target population. Participants aged 12–17 years had to have ≥10 kidney cysts, each measuring ≥0.5 cm on magnetic resonance imaging (MRI). Those aged 4–11 years did not undergo MRI and had to have ≥4 kidney cysts on ultrasound, each measuring ≥1 cm.

Exclusion criteria included alanine aminotransferase/aspartate aminotransferase ≥1.5 × upper limit of normal or any medical condition that could interfere with the evaluation of the trial objectives or endanger participant safety.

### Trial Design

This was a phase 3b, two-part trial (EudraCT number: 2016-000187-42; ClinicalTrials.gov identifier: NCT02964273). Phase A was a 1-year, randomized, double-blind, placebo-controlled, multicenter trial; phase B is an ongoing, 2-year, open-label extension. The phase A population was randomized to tolvaptan or a matching placebo in a 1:1 ratio (see the Supplemental Methods for the randomization procedure).

### Treatments

Participants received tolvaptan or placebo daily in a split-dose regimen, with the first dose taken upon waking and the second 8–9 hours later (Supplemental Methods). Starting doses were based on body weight (Supplemental Table 1). After 1 week, participants who tolerated the initial dose up-titrated once to a maximum dose that was dependent on body weight and lower than in the adult studies in the expectation that a trial population with well-preserved kidney function will respond to tolvaptan with potent diuresis. The daily starting dose and up-titrated maximum dose, respectively, were 15/7.5 and 30/15 mg for participants ≥20 to <45 kg, 30/15 and 45/15 mg for participants ≥45 to ≤75 kg, and 45/15 and 60/30 mg for participants >75 kg. Participants could down-titrate at any time on the basis of tolerability but were asked to stay on the highest tolerated dose.^21^

### End Points

Given that treatment effects on TKV or kidney function are difficult to measure in a pediatric population with early-stage disease, pharmacodynamic parameters were considered the most appropriate coprimary end points: change from baseline in spot urine osmolality and spot urine specific gravity at week 1 in all participants. Spot urine samples were collected before the morning dose and before eating or drinking anything for breakfast from the urine void (second-morning void) taken after the first morning's void and ideally collected as a mid-stream, clean catch. The samples were analyzed as described in the Supplemental Methods. The key secondary end point was percent change in height-adjusted TKV (htTKV) from baseline to month 12 in group 1; volumetric measurement was performed in this group using MRI. Percent change in htTKV on ultrasound from baseline to month 12 was evaluated for group 2 as an exploratory end point. An additional secondary end point was change from baseline in kidney function (eGFR by bedside Schwartz formula).^22^ Safety and tolerability were secondary end points, including changes from baseline in creatinine, vital signs, laboratory values, urine volume, and adverse events (AEs). Quality of life on generic pediatric quality-of-life assessment instruments was an exploratory end point.

### Assessments

Postscreening assessment visits occurred at baseline (day 1), week 1, and monthly for 12 months. Participants aged 12–17 years underwent TKV assessment by MRI performed according to published standards at screening and month 12.^12^ Scans were read at a central laboratory by individuals blinded to treatment assignment. Participants aged <12 years underwent ultrasound at screening and month 12, with TKV estimated using an ellipsoid volume equation with three axes of measurement.^23,24^ Health-related quality of life was assessed using the PedsQL Generic Core Scale (which measures four dimensions of function: physical, emotional, social, and school-related) and Multidimensional Fatigue Scale (which measures three dimensions of fatigue: general, sleep/rest-related, and cognitive) at baseline, week 1, and months 1, 3, 6, and 12.^25–27^ Both scales range from 0 to 100, with higher scores indicating better quality of life. Additional information on assessments is presented in the Supplemental Methods.

### Statistical Analyses

A sample size of ≥60 participants aged 12–17 years was planned. It was expected that approximately 40 participants aged 4–11 years would also enroll for an anticipated total enrollment of 100 participants. As data were to be summarized descriptively, no formal power calculations to determine sample size were undertaken. All outcomes, including primary and secondary outcomes, were evaluated using observed values only (no missing data imputation). Missing data were to be mitigated by encouraging participants who withdrew from treatment to return for their remaining visits.

The full analysis set, which was a modified intent-to-treat population, was used for the efficacy analyses. It consisted of all participants who were randomized to a treatment group, received ≥1 dose of the trial drug, and had both a phase A baseline and ≥1 postbaseline efficacy evaluation. The safety population consisted of all participants who received ≥1 dose of the trial drug. As the study was designed without formal efficacy testing, statistical comparisons between treatment groups (described in the Supplemental Methods) were *post hoc* and exploratory.

## Results

### Participant Disposition

**Figure 1:**
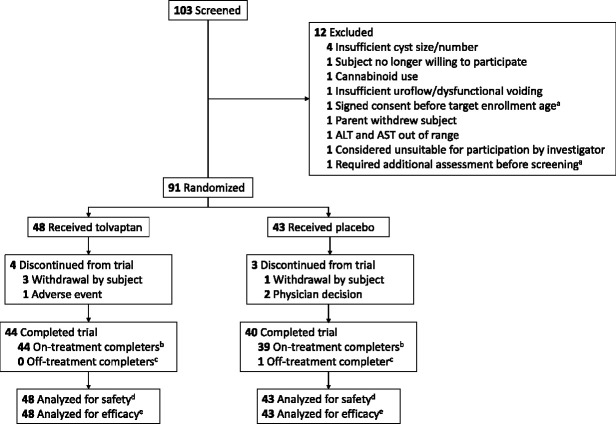
**Participant disposition.** ^a^The participant was later rescreened and entered the study. ^b^Randomized participants who completed the month 12 visit on study medication. ^c^Randomized participants who discontinued study medication before the month 12 visit but continued with trial visit assessments. ^d^All participants who were randomized and took ≥1 dose of study medication after randomization. ^e^All participants who were in the randomized population, took ≥1 dose of study medication after randomization, and had a baseline and ≥1 valid postbaseline efficacy evaluation. ALT, alanine aminotransferase; AST, aspartate aminotransferase.

Ninety-one participants were randomized (Figure 1), including 66 in the 12- to 17-year-old cohort (group 1) and 25 in the 4- to 11-year-old cohort (group 2). All randomized participants took ≥1 dose of the study drug and had a baseline and ≥1 postbaseline efficacy evaluation and were therefore included in the safety and primary efficacy analyses. Among all randomized participants, 84 (92%) completed the 1-year, randomized, double-blind phase (phase A) and seven (8%) discontinued. One participant completed phase A while not on treatment (*i.e.*, discontinued trial medication [placebo] before the month 12 visit but continued with trial visit assessments). All eight participants who discontinued from the study or treatment were from group 1. Participant disposition by age subgroup is shown in Supplemental Figure 1. Phase A concluded in December 2019.

### Baseline Characteristics

Demographic characteristics in the total population were similar across treatment arms (Table 1). The most frequently reported ADPKD-related medical histories were hypertension (*n*=21/91 [23%]) and proteinuria (*n*=14/91 [15%]). One participant in the tolvaptan arm had a concomitant diagnosis of tuberous sclerosis and hence a notably larger kidney size than the other participants. Baseline characteristics are shown by age subgroup in Supplemental Table 2.

**Table 1:** Baseline demographic and clinical characteristics of children and adolescents enrolled in a randomized, placebo-controlled clinical trial of tolvaptan

Characteristic	Tolvaptan (*n*=48)	Placebo (*n*=43)	Total (*n*=91)
**Age (yr)**			
Mean (SD)	12.9 (3.2)	12.8 (2.8)	12.9 (3.0)
Range	5–17	6–17	5–17
**Age subgroup, *n* (%)**			
<12 yr	13 (27)	12 (28)	25 (27)
12–14 yr	17 (35)	15 (35)	32 (35)
15–17 yr	18 (38)	16 (37)	34 (37)
Female, *n* (%)	21 (44)	23 (54)	44 (48)
**Race, *n* (%)**			
White	46 (96)	42 (98)	88 (97)
Black	0	1 (2)	1 (1)
Asian	2 (4)	0	2 (2)
**Ethnicity, *n* (%)**			
Hispanic or Latino	1 (2)	1 (2)	2 (2)
Not Hispanic or Latino	47 (98)	42 (98)	89 (98)
**Weight (kg)**			
Mean (SD)	54 (16)	51 (18)	53 (17)
Range	21–79	23–108	21–108
**Height (cm)**			
Mean (SD)	161 (18)	159 (16)	160 (17)
Range	113–193	115–186	113–193
**Body mass index (kg/m** ^**2**^ **)**			
Mean (SD)	20.4 (3.7)	19.6 (4.1)	20.0 (3.9)
Range	14.2–28.9	15.0–34.9	14.2–34.9
Growth percentile, mean (SD)	63 (26)	67 (24)	65 (25)
**Diagnosis age (yr)**			
Mean (SD)	5.2 (5.2)	7.7 (5.4)	6.4 (5.4)
Range	0–17	0–16	0–17
Genetic testing performed, *n* (%)	14 (29)	13 (30)	27 (30)
**Other blood-related family with PKD, (%)**			
Yes	43 (90)	39 (91)	82 (90)
No	4 (8)	4 (9)	8 (9)
Unknown	1 (2)	0	1 (1)
Aware of family history before diagnosis, *n* (%)	43 (90)	39 (91)	82 (90)
**Reason for diagnosis, *n* (%)**			
Consequence of ADPKD signs or symptoms	6 (13)	17 (40)	23 (25)
Incidental (because of tests unrelated to ADPKD or its symptoms)	10 (21)	5 (12)	15 (17)
Asymptomatic screening (no prior ADPKD symptoms)	32 (67)	20 (47)	52 (57)
Spot urine osmolality (mOsm/kg), mean (SD)	635 (252)	646 (250)	640 (249)
Urine specific gravity, mean (SD)	1.017 (0.006)	1.017 (0.006)	1.017 (0.006)
**htTKV (ml/cm)**			
Aged 12–17 yr, *n*	30	27	57
*Mean (SD)*	3.5 (4.29)	2.7 (0.79)	3.1 (3.16)
Aged 4–11 yr, *n*	9	6	15
*Mean (SD)*	1.7 (0.72)	2.8 (1.68)	2.2 (1.27)
eGFR by bedside Schwartz formula, ml/min per 1.73 m^2^, mean (SD)	99 (19.4)	100 (15.0)	99 (17.4)
**ADPKD medical history, *n* (%)** ^a^			
Hepatic cysts	4 (8)	2 (5)	6 (7)
Non–hepato-kidney cysts	1 (2)	0	1 (1)
Gross hematuria	3 (6)	0	3 (3)
Upper urinary tract infection	1 (2)	3 (7)	4 (4)
Proteinuria	7 (15)	7 (16)	14 (15)
Hypertension	7 (15)	14 (33)	21 (23)
Kidney pain	5 (10)	6 (14)	11 (12)

PKD, polycystic kidney disease; ADPKD, autosomal dominant polycystic kidney disease; htTKV, height-adjusted total kidney volume.

a There were no participants with a medical history of nephrolithiasis, anemia, colonic diverticulitis, or vascular/cardiac abnormalities.

### Exposure to Study Medication

In all randomized participants, exposure of ≥361 days was achieved in 33 of 48 participants (69%) in the tolvaptan arm and 29 of 43 participants (67%) in the placebo arm. The average daily dose was 41 (SD 17) mg in the tolvaptan arm and 51 (SD 14) mg in the placebo arm. The most frequent modal total daily doses at month 12 were 45 mg for tolvaptan (39% participants) and 60 mg for placebo (53% participants) in group 1 and 15 mg and 22.5 mg (31% participants each) for tolvaptan and 45 mg (67% participants) for placebo in group 2 (Supplemental Table 3).

Treatment adherence in all participants, as determined by returned study medication, was ≥90% for 46 of 48 participants (96%) in the tolvaptan arm and 40 of 43 participants (93%) in the placebo group. Plasma concentrations of the tolvaptan oxobutyric acid metabolite (DM-4103) were used to verify adherence in tolvaptan-treated participants. DM-4103 concentrations at week 1 and month 1 were consistent with reported dosing for all participants with an evaluable sample. At month 6, one of 42 participants (2%) with a pharmacokinetic sample was not adherent with reported dosing, and at month 12, five additional participants (for a total of six of 40 participants [15%]) had DM-4103 concentrations indicating limited to no dosing within the previous weeks. Of these six nonadherent participants, five participants were from group 1 and one participant was from group 2.

### Pharmacodynamics

For the first coprimary efficacy end point (all participants), least squares (LS) mean (±SEM) reduction from baseline in spot urine osmolality (premorning dose) after 1 week of daily dosing was greater in the tolvaptan arm (−390 [28] mOsm/kg) than in the placebo arm (−90 [29] mOsm/kg; *P*<0.001; Table 2). For the second coprimary efficacy end point, LS mean reduction from baseline in urine specific gravity (premorning dose) after 1 week was greater in the tolvaptan group (−0.009 [0.001]) versus the placebo group (−0.002 [0.001]; *P*<0.001). The results were comparable within each age subgroup (Supplemental Figure 2).

**Table 2:** Coprimary, key secondary, and related outcomes

Outcome and Treatment Assignment	Value at Baseline		Value at Follow-Up		Change from Baseline	Difference in Change (95% CI)	*P* Value
	*n* ^a^	Mean (SD)	*n* ^a^	Mean (SD)	LS Mean (SEM)		
**Change in spot urine osmolality (mOsm/kg) at week 1, full analysis set (coprimary)**							
Tolvaptan	48	635 (252)	48	250 (142)	−390 (28)	−301 (−381 to −220)	<0.001
Placebo	43	646 (250)	42	553 (234)	−90 (29)		
**Change in spot urine osmolality (mOsm/kg) at month 1, full analysis set**							
Tolvaptan	48	635 (252)	48	275 (169)	−363 (33)	−320 (−416 to −224)	<0.001
Placebo	43	646 (250)	43	598 (307)	−44 (35)		
**Change in urine specific gravity at week 1, full analysis set (coprimary)**							
Tolvaptan	48	1.017 (0.006)	48	1.008 (0.004)	−0.009 (0.001)	−0.007 (−0.009 to −0.005)	<0.001
Placebo	43	1.017 (0.006)	41	1.015 (0.006)	−0.002 (0.001)		
**Change in urine specific gravity at month 1, full analysis set**							
Tolvaptan	48	1.017 (0.006)	48	1.009 (0.004)	−0.009 (0.001)	−0.008 (−0.011 to −0.006)	<0.001
Placebo	43	1.017 (0.006)	43	1.017 (0.008)	0.000 (0.001)		
**Change in htTKV (ml/cm) on MRI at month 12, ages 12–17 yr (key secondary)**							
Tolvaptan	30	3.5 (4.3)	30	3.5 (3.7)	2.6% (1.4)	−3.2% (−7.4 to 1.0)	0.14
Placebo	27	2.7 (0.8)	27	2.8 (0.8)	5.8% (1.5)		
**Change in htTKV (ml/cm) on MRI at month 12, ages 12–17 yr excluding htTKV outlier** **^b^**							
Tolvaptan	29	2.7 (1.1)	29	2.8 (1.2)	2.9% (1.5)	−3.2% (−7.4 to 1.1)	0.14
Placebo	27	2.7 (0.8)	27	2.8 (0.8)	6.1% (1.5)	
**Change in htTKV (ml/cm) on MRI at month 12, ages 12–17 yr excluding tolvaptan nonadherent** **^c^**							
Tolvaptan	26	3.5 (4.6)	26	3.5 (3.9)	2.3% (1.6)	−3.5% (−7.9 to 0.9)	0.12
Placebo	27	2.7 (0.8)	27	2.8 (0.8)	5.8% (1.5)	

LS, least squares; 95% CI, 95% confidence interval; MRI, magnetic resonance imaging; htTKV, height-adjusted total kidney volume.

a For the baseline visit, *n* is the total number of treated participants with a predose evaluation. For postdose visits, *n* is the total number of treated participants with both baseline and postdose evaluations at the specific visit.

b A participant with tuberous sclerosis and hence notably larger kidney volume was excluded.

c Participants with pharmacokinetically verified nonadherence were excluded.

### Efficacy

**Figure 2:**
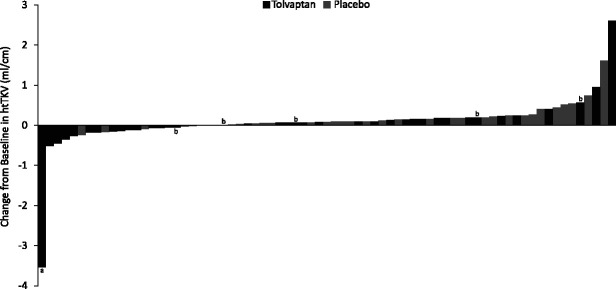
**Waterfall plot of change from baseline to month 12 in height-adjusted total kidney volume (htTKV) for individual participants (all participants).** ^a^The kidney volume outlier with tuberous sclerosis. ^b^Participant with pharmacokinetically verified nonadherence with tolvaptan treatment.

The LS mean (±SEM) percent htTKV increase on MRI in group 1 at month 12, the key secondary study end point, was 2.6% (1.4) with tolvaptan and 5.8% (1.5) with placebo (*P*=0.14; Table 2). Other analyses of htTKV were conducted and are also shown in Table 2. The mean percent increase in htTKV from baseline to month 12 for the overall study population is shown in Supplemental Figure 3, and changes in htTKV to month 12 for the overall population are displayed by individual in Figure 2. Supplemental Figure 4 shows the prespecified exploratory end point of LS mean (±SEM) percent increase from baseline to month 12 in ultrasound htTKV for group 2 (17.2% [20.0] for tolvaptan versus 21.1% [25.0] for placebo; *P*=0.91).

**Figure 3:**
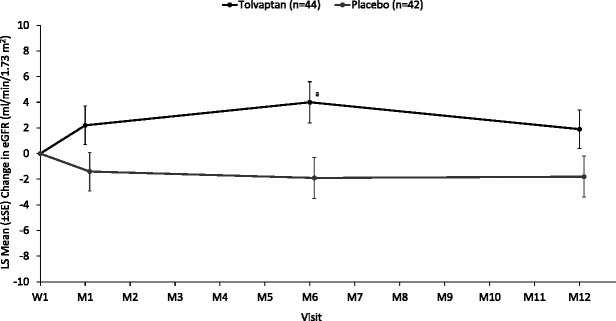
**Secondary end point: change in eGFR from week 1 through month 12 (all participants).** ^a^*P*<0.05 for tolvaptan versus placebo. LS, least squares; M, month; W, week.

Baseline eGFR (±SD) in all study participants was 99 (19) ml/min per 1.73 m^2^ for the tolvaptan group and 100 (15) ml/min per 1.73 m^2^ for the placebo group. To exclude the known hemodynamic effect of tolvaptan and show the longer-term effects of treatment, the secondary end point of change in eGFR is provided from week 1 to month 12 for all participants (Figure 3). The LS mean (±SEM) change in eGFR from week 1 to month 12 was 1.9 (1.5) ml/min per 1.73 m^2^ for the tolvaptan arm and −1.8 (1.6) ml/min per 1.73 m^2^ for the placebo arm (*P*=0.11 for between-treatment difference). Other analyses of eGFR were conducted and are shown in Supplemental Figures 5–7.

### Safety and Tolerability

Treatment-emergent AEs were reported for 44 of 48 participants (92%) in the tolvaptan group and 42 of 43 participants (98%) in the placebo group during phase A of the trial. The most frequent AEs are shown in Table 3 and, per protocol, were based on investigator reporting rather than prespecified criteria. The incidence of aquaretic treatment-emergent AEs (Medical Dictionary for Regulatory Activities [MedDRA] preferred terms dry mouth, thirst, polydipsia, nocturia, pollakiuria, and polyuria) was higher in the tolvaptan group (31 of 48 participants [65%]) than in the placebo group (seven of 43 participants [16%]). Dehydration-related treatment-emergent AEs, which included all preferred terms under the dehydration standardized MedDRA queries^30^ that were reported in this trial (tachycardia, dry mouth, lip dry, thirst, blood sodium increased, dehydration, hypernatremia, hypotension, and orthostatic hypotension), also occurred more frequently in the tolvaptan group (17 of 48 participants [35%]) than in the placebo group (six of 43 participants [14%]). Investigator-reported serum sodium increases occurred in two of 48 tolvaptan-treated participants (4.2%) and zero participants who received placebo, and investigator-reported hypernatremia occurred in one of 48 tolvaptan-treated participants (2.1%) and zero participants who received placebo. By predefined laboratory criteria, there were no events of potentially clinically significant increases or decreases in sodium in either treatment group. One tolvaptan-treated participant experienced a potentially clinically significant increase in potassium (from 4.2 mEq/L at baseline to 5.7 mEq/L at month 12), and none had a potentially clinically significant decrease. The definitions of potentially clinically significant changes in laboratory parameters are provided in Supplemental Table 4. AEs by age subgroup and in tolvaptan-adherent participants are shown in Supplemental Tables 5 and 6.

**Table 3:** Summary of adverse events and listing of individual treatment-emergent adverse events (by system organ class and MedDRA preferred term) occurring in ≥5% of participants in either treatment group

*n* (%)	Tolvaptan (*n*=48)	Placebo (*n*=43)
Participant days of drug exposure	16,502	15,060
Participants with AEs	45 (94)	42 (98)
AEs	344	307
Participants with treatment-emergent AEs^a^	44 (92)	42 (98)
Treatment-emergent AEs	289	237
Participants with serious treatment-emergent AEs	1 (2)	6 (14)
Participants with nonserious treatment-emergent AEs	44 (92)	42 (98)
Participants with severe treatment-emergent AEs	1 (2)	2 (5)
Participants discontinued study medication because of AEs	1 (2)	1 (2)^b^
Deaths	0	0
**Ear and labyrinth disorders**		
Ear pain	3 (6)	2 (5)
**Gastrointestinal disorders**		
Abdominal pain	6 (13)	3 (7)
Abdominal pain upper	5 (10)	4 (9)
Constipation	5 (10)	1 (2)
Diarrhea	3 (6)	7 (16)
Nausea	3 (6)	7 (16)
Vomiting	7 (15)	10 (23)
**General disorders and administration site conditions**		
Fatigue	4 (8)	3 (7)
Pyrexia	4 (8)	3 (7)
Thirst	7 (15)	2 (5)
**Immune system disorders**		
Seasonal allergy	4 (8)	1 (2)
**Infections and infestations**		
Bronchitis	3 (6)	1 (2)
Ear infection	1 (2)	4 (9)
Nasopharyngitis	10 (21)	14 (33)
Pharyngitis	4 (8)	0
Rhinitis	3 (6)	3 (7)
Upper respiratory tract infection	4 (8)	2 (5)
Viral infection	3 (6)	1 (2)
**Investigations**		
Serum creatinine increased	9 (19)	2 (5)
**Metabolism and nutrition disorders**		
Decreased appetite	4 (8)	2 (5)
Polydipsia	5 (10)	1 (2)
**Musculoskeletal and connective tissue disorders**		
Back pain	4 (8)	5 (12)
Pain in extremity	0	6 (14)
**Nervous system disorders**		
Dizziness	3 (6)	5 (12)
Headache	16 (33)	21 (49)
Migraine	2 (4)	3 (7)
**Kidney and urinary disorders**		
Nocturia	7 (15)	3 (7)
Pollakiuria	9 (19)	0
Polyuria	13 (27)	2 (5)
Kidney pain	2 (4)	3 (7)
**Respiratory, thoracic, and mediastinal disorders**		
Cough	7 (15)	5 (12)
Epistaxis	3 (6)	0
Oropharyngeal pain	4 (8)	6 (14)
**Vascular disorders**		
Hypertension	4 (8)	1 (2)
Orthostatic hypotension	5 (10)	0

AE, adverse event.

a Defined as an AE that started after the initiation of study medication; or if the event was continuous from baseline and was serious, study medication-related, or resulted in death, discontinuation, interruption, or reduction of study medication. Multiple occurrences of treatment-emergent AEs are counted once per MedDRA preferred term.

b This participant discontinued trial medication but continued participating in the trial (*i.e.*, attended trial visits).

Data on urine volume in the 24 hours after dosing were collected for a subset of tolvaptan (*n*=12) and placebo (*n*=8) participants aged 12–17 years who participated in dense pharmacokinetic/pharmacodynamic sampling after at least 1 month on treatment. Urine volume (±SD) in tolvaptan-treated participants (7171 [2810] ml) was substantially higher than in participants receiving placebo (2529 [2164] ml). Higher urine volume was reflected in the number of voids during the day and night among tolvaptan-treated participants (Supplemental Figure 8). The tolvaptan-treated participants experienced an initial peak in daytime and nighttime voids at week 1 of treatment and a decline thereafter. No indication of urinary or bladder-related AEs was observed.

In the nine tolvaptan-treated participants who experienced increased creatinine, the event resolved in all patients, with no action taken regarding tolvaptan in six participants, tolvaptan interruption in two participants, and tolvaptan dose reduction in one participant. Potentially clinically significant increases in creatinine occurred in four participants in the tolvaptan group and no participant in the placebo group during phase A.

For five hepatic standardized MedDRA queries (cholestasis and jaundice of hepatic origin; hepatic failure, fibrosis and cirrhosis, and other liver damage-related conditions; noninfectious hepatitis; liver-related investigations, signs, and symptoms; and liver-related coagulation and bleeding disturbances),^30^ there was one treatment-emergent AE (increased blood bilirubin) in the placebo group during phase A. No participant experienced elevated transaminases, and there were no cases of drug-induced liver injury.

One participant in the tolvaptan group had a serious treatment-emergent AE (viral pericarditis). Six participants in the placebo group had serious treatment-emergent AEs: one had a hand fracture and ulna fracture, one had hematuria and kidney pain, one had an intentional overdose and intentional self-injury, and one participant each had petit mal epilepsy, hypertensive crisis, and pelvic pain. None of the serious treatment-emergent AEs reported in either treatment group were assessed by the investigator as related to the study drug. There were no deaths. One participant in the tolvaptan group and two participants in the placebo group had treatment-emergent AEs that were assessed as severe (Supplemental Material).

Two participants discontinued treatment because of AEs: one in the tolvaptan group (because of pollakiuria) and one in the placebo group (because of dizziness). Both events were assessed as of moderate severity and related to the study drug.

Mean changes from baseline in height SD scores were similar between the tolvaptan and placebo arms across age and sex subgroups (Supplemental Table 7). There were no notable differences in Tanner staging progression between treatment groups during the trial (Supplemental Table 8).

The PedsQL Generic Core Scale and the PedsQL Multidimensional Fatigue Scale showed that quality of life was unchanged from baseline to month 12 (Supplemental Table 9).

## Discussion

We report the first phase 3, interventional clinical trial of tolvaptan in children and adolescents with ADPKD. The current report focuses on the randomized, placebo-controlled, 1-year portion (phase A) of the study. In the absence of standardized criteria for identifying the potential for rapid progression in the pediatric ADPKD population,^31^ enrollment criteria were developed based on expert consensus, taking into account family history, genetic data, and cyst burden.^21^ The trial was designed to provide descriptive data, and all statistical comparisons of the treatment groups reported here were performed as exploratory analyses.

Changes from baseline in spot urine osmolality and specific gravity were greater in the tolvaptan group than in the placebo group, with results consistent across age groups. The observation of more dilute urine is supportive of vasopressin V2 receptor inhibition. The key secondary end point of percent change in htTKV from baseline to month 12 showed that participants aged 12–17 years at baseline experienced a lower rate of kidney volume growth with tolvaptan (2.6%) than placebo (5.8%), although the difference did not reach statistical significance. Similarly, the change in eGFR from week 1 to month 12 suggested that eGFR was preserved in the tolvaptan group relative to placebo. The exploratory statistical comparison did not show a significant effect of tolvaptan at month 12. The mean eGFR at baseline and at month 12 was >90 ml/min per 1.73 m^2^ in each treatment group; as such, the assessment of changes in eGFR at this early stage of disease progression was difficult. Despite these caveats, the hypothesis that tolvaptan may slow kidney function decline in children with ADPKD because of inhibition of cystic growth and the attendant damage to kidney parenchyma is plausible and merits further evaluation. Significant between-treatment differences in eGFR change were observed at months 1 and 6 in the adolescent participants (group 1) and at month 12 in children aged 4–11 years (group 2; Supplemental Figures 6 and 7). Of note, potential effects on eGFR were observed in a study population receiving modal total daily doses of tolvaptan that were reduced relative to adult doses, with most tolvaptan-treated participants taking less than the minimum adult dose (*i.e.*, 60 mg/d).

Tolvaptan was generally safe and well tolerated at the adjusted doses used in this trial. No participants experienced elevated transaminases or drug-induced liver injury. Events of increased blood creatinine and orthostatic hypotension in the tolvaptan group may have been effects of copious aquaresis with inadequate fluid replacement. Consistent with its mechanism of inhibiting the effects of antidiuretic hormone, tolvaptan was associated with aquaretic adverse effects in this population with high kidney function, increased urine volume, and an increased number of urine voids. The number of voids was highest at week 1 of treatment and declined thereafter. Despite the high mean 24-hour urine volume (>7000 ml) in the tolvaptan-treated subgroup evaluated—exceeding levels observed in adults^32^—and the related increase in number of voids, few participants (*n*=4) discontinued tolvaptan. This finding suggests that tolvaptan-related aquaretic AEs were manageable, especially as participants could down-titrate tolvaptan if needed for tolerability. Patient acceptance of aquaretic effects is supported by PedsQL scores, which did not show deterioration in quality of life. Discontinuations and nonadherence with tolvaptan were almost entirely in the older group (aged 12–17 years), possibly because of greater involvement of parents of the younger group (aged 4–11 years) in study participation. Although the age subgroups analyzed were small, changes from baseline in height SD scores raised no growth-related concerns for tolvaptan treatment relative to placebo.

A limitation of this study is that it was not powered for the statistical comparison of htTKV and eGFR end points. The first study phase, as reported here, was only 12 months, further limiting conclusions regarding these end points. Given the challenges of identifying sufficient numbers of pediatric patients with ADPKD for clinical trials,^11^ the sample size was relatively small, and the trial design could not include the calculation of statistical power for efficacy end points. A larger, genetically well-characterized population with more defined scoring will be needed for future pediatric studies.^33^ Missing efficacy data were not imputed, further limiting the size of the analysis populations for the efficacy end points.

The data here support the activity of tolvaptan at the vasopressin V2 receptor in children and adolescents with ADPKD, indicate the feasibility of a modified dosing strategy, and provide initial evidence of effects on TKV growth and eGFR decline that require further research. This trial provides information beyond case reporting on the use of tolvaptan in an infant ADPKD patient.^34^ The open-label extension phase is ongoing and will generate longer-term follow-up data.

## Supplementary Material

## Data Availability

To submit inquiries related to Otsuka clinical research, or to request access to individual participant data (IPD) associated with any Otsuka clinical trial, please visit https://clinical-trials.otsuka.com/. For all approved IPD access requests, Otsuka will share anonymized IPD on a remotely accessible data-sharing platform.

## Supplemental Material

This article contains the following supplemental material online at http://links.lww.com/CJN/B82. Supplemental Methods. Severe Adverse Events. Supplemental Table 1. Up-titration and down-titration steps. Supplemental Table 2. Baseline demographic and clinical characteristics in participants aged 12–17 years (group 1) and participants aged 4–11 years (group 2). Supplemental Table 3. Modal total daily dose at study visits. Supplemental Table 4. Criteria for potentially clinically significant changes in laboratory values. Supplemental Table 5. Summary of adverse events and listing of individual treatment-emergent adverse events (by system organ class and MedDRA preferred term) occurring in ≥10% of participants in any treatment arm, participants aged 12–17 years (group 1) and 4–11 years (group 2). Supplemental Table 6. Summary of adverse events and listing of individual treatment-emergent adverse events (by system organ class and MedDRA preferred term) occurring in ≥5% of tolvaptan-treated participants, full safety population, and excluding participants with verified nonadherence. Supplemental Table 7. Mean change from baseline in height SD scores (SDS) by age and sex (reference: Northern European populations). Supplemental Table 8. Tanner staging at baseline and month 12 in participants aged 12–17 years (group 1) and 4–11 years (group 2). Supplemental Table 9. Scores on the Pediatric Quality of Life Inventory (PedsQL) Acute Version 4.0 Generic Core Scale and PedsQL Multidimensional Fatigue Scale Acute Version 3.0. Supplemental Figure 1. Participant disposition by age group. Supplemental Figure 2. Change from baseline in spot urine osmolality (mOsm/kg) and urine specific gravity in (A) participants aged 12–17 years (group 1) and (B) participants aged 4–11 years (group 2). Supplemental Figure 3. Percent change from baseline to month 12 in htTKV on MRI (in participants aged 12–17 years) and ultrasound (in participants aged 4–11 years), all participants. Supplemental Figure 4. Percent change from baseline to month 12 in htTKV on ultrasound in participants aged 4–11 years (group 2). Supplemental Figure 5. Change in eGFR from week 1 through month 12, all participants excluding tolvaptan participants with verified nonadherence. Supplemental Figure 6. Change in eGFR from week 1 through month 12, participants aged 12–17 years (group 1). Supplemental Figure 7. Change in eGFR from week 1 through month 12, participants aged 4–11 years (group 2). Supplemental Figure 8. Mean daytime and nighttime number of voids from participants aged 12–17 years (group 1).
