# Supplementary material for: Tolvaptan for Children and Adolescents with Autosomal Dominant Polycystic Kidney Disease: Randomized Controlled Trial
Source: Clin J Am Soc Nephrol. 2022 Jan 18;18(1):36–46. doi: 10.2215/CJN.0000000000000022 (PMC10101612; doi:10.2215/CJN.0000000000000022)
Supplement: Supplementary file 1 [file cjasn-18-036-s001.pdf]

Supplemental Appendix

Supplemental Methods

Severe Adverse Events

Supplemental Table 1. Up-titration and down-titration steps

Supplemental Table 2. Baseline demographic and clinical characteristics in participants aged 12–17 years (Group 1) and participants aged 4–11 years (Group 2)

Supplemental Table 3. Modal total daily dose at study visits

Supplemental Table 4. Criteria for potentially clinically significant changes in laboratory values

Supplemental Table 5. Summary of adverse events and listing of individual treatment-emergent adverse events (by system organ class and MedDRA preferred term) occurring in ≥10% of participants in any treatment arm, participants aged 12–17 years (Group 1) and 4–11 years (Group 2)

Supplemental Table 6. Summary of adverse events and listing of individual treatment-emergent adverse events (by system organ class and MedDRA preferred term) occurring in ≥5% of tolvaptan-treated participants, full safety population and excluding participants with verified nonadherence

Supplemental Table 7. Mean change from baseline in height standard deviation scores (SDS) by age and sex (reference: Northern European populations)

Supplemental Table 8. Tanner staging at baseline and Month 12 in participants aged 12–17 years (Group 1) and 4–11 years (Group 2)

Supplemental Table 9. Scores on the Pediatric Quality of Life Inventory (PedsQL™) Acute Version 4.0 Generic Core Scale and PedsQL™ Multidimensional Fatigue Scale Acute Version 3.0

Supplemental Figure 1. Participant disposition by age group

Supplemental Figure 2. Change from baseline in spot urine osmolality (mOsm/kg) and urine specific gravity in (A) participants aged 12–17 years (Group 1) and (B) participants aged 4–11 years (Group 2)

Supplemental Figure 3. Percent change from baseline to Month 12 in htTKV on MRI (in participants aged 12–17 years) and ultrasound (in participants aged 4–11 years), all participants

Supplemental Figure 4. Percent change from baseline to Month 12 in htTKV on ultrasound in participants aged 4–11 years (Group 2)

Supplemental Figure 5. Change in eGFR from Week 1 through Month 12, all participants excluding tolvaptan participants with verified nonadherence

Supplemental Figure 6. Change in eGFR from Week 1 through Month 12, participants aged 12–17 years (Group 1)

Supplemental Figure 7. Change in eGFR from Week 1 through Month 12, participants aged 4–11 years (Group 2)

Supplemental Figure 8. Mean daytime and nighttime number of voids from in participants aged 12–17 years (Group 1)

Supplemental Methods

Ethics approval

The trial was conducted in compliance with FDA regulations, International Conference on Harmonization Good Clinical Practice Guideline (E6), international ethical principles derived from the Declaration of Helsinki, Council for International Organizations of Medical Science guidelines, and applicable local laws and regulations. Copies of the protocol, amendments, and the informed consent form/informed assent form were reviewed and approved by the governing independent ethics committee for each investigational site or country, as appropriate, prior to trial start or prior to implementation of the protocol or protocol amendment at that site and/or country.

Consent to participate

Age-appropriate assent documents were created and received institutional review board/independent ethics committee approval. Written informed consent was obtained from all participants and from participants' guardians or legally acceptable representatives, as applicable by local laws. Participants who became legal adults during the trial were required to provide written informed consent as soon as they reached legal age.

Randomization procedure

The target population of ≥60 participants aged 12–17 years was stratified by gender and age into 4 cohorts of approximately 15 participants each: males and females 15–17 years and males and females 12–14 years. Randomization was performed according to a computer-generated randomization schedule supplied by the sponsor to the treatment-blinded investigators and site staff. When >15 participants were enrolled in a cohort, new screening for that cohort was closed; participants concurrently in screening for that cohort could be enrolled if they met inclusion/exclusion criteria. When the last participant in the final cohort for the required population was achieved, enrollment in the trial ended.

Treatments

Tolvaptan/placebo was administered with a recommended 240 mL of water within a 1-hour period. Participants were also encouraged to drink plain water per thirst throughout the day and one to two glasses of water before bedtime to help maintain proper hydration status.

Timing of assessments

Spot urine osmolality and spot urine specific gravity (co-primary endpoints) were assessed at baseline, Week 1, and Month 1. Serum creatinine and estimated glomerular filtration rate (eGFR) were assessed at all visits, and serum sodium was assessed at all visits until Month 11. Sparse pharmacokinetic blood samples were collected from all participants at Week 1 and Months 1, 6, and 12 for determination of plasma tolvaptan and its metabolite concentrations. As tolvaptan has aquaretic effects, urinary parameters were evaluated. The number of daytime and nighttime voids over 24 hours was recorded at baseline, Week 1, and Months 1, 6, and 12.

At each trial visit, the investigator elicited adverse events with a nonleading question. Participants were tested for alanine aminotransferase (ALT), aspartate aminotransferase (AST), alkaline phosphatase, and total bilirubin at screening/baseline and for ALT and AST at Week 1 and every month thereafter.

Analysis of urine samples for co-primary endpoints

Urine osmolality and specific gravity were analyzed at Covance Central Laboratory Services, Geneva, Switzerland (now Labcorp Drug Development). Urine osmolality was assessed using freezing point

depression and specific gravity using a reagent test strip with automated reader. Further details would be available from the vendor.

Statistical analyses

Comparisons for the co-primary endpoints and eGFR were conducted using the mixed model repeated measures method with fixed effects of treatment, visit, treatment by visit interaction, baseline, and baseline by visit interaction as covariates, with an unstructured variance covariance structure. Least-squares (LS) mean values for each treatment arm were compared at each time point. For the key secondary endpoint and other height-adjusted total kidney volume outcomes, analysis of covariance was used, with baseline value as a covariate and treatment as a main effect. Comparisons for these outcomes were based on LS mean values at Month 12.

Severe Adverse Events

Severe adverse events were those which were assessed by the investigator as causing inability to work or perform normal daily activity. In the tolvaptan group, one participant had severe polydipsia and polyuria, both of which were assessed by the investigator as related to the study drug. In the placebo group, one participant had severe hematuria and kidney pain, and one participant had severe hand fracture and ulna fracture; none of these events were assessed as related to the study drug.

Supplemental Table 1. Up-titration and down-titration steps*

**Up-titration Steps**		
**Body Weight**	**Starting Dose**	**Up-titrated Maximum Dose**
≥20 kg to <45 kg	15/7.5 mg	30/15 mg
≥45 kg to ≤75 kg	30/15 mg	45/15 mg
>75 kg	45/15 mg	60/30 mg

**Down-titration Steps**	
**Current Dose**	**Down-titration Steps**
7.5 mg once daily upon awakening	Participant discontinues treatment
7.5/7.5 mg	7.5 mg once daily upon awakening
15/7.5 mg	7.5/7.5 mg
22.5/15 mg	15/7.5 mg
30/15 mg	22.5/15 mg
45/15 mg	30/15 mg
60/30 mg	45/15 mg

*Tolvaptan or placebo.

Children weighing <45 kg and 45–75 kg had up-titrated doses that were 37.5% and 50%, respectively, of the highest recommended total adult target dose (90/30 mg/day). The up-titrated dose in children and adolescents weighing >75 kg was 75% of the highest recommended adult target dose. Adults are up-titrated in 3 steps.

Supplemental Table 2. Baseline demographic and clinical characteristics in participants aged 12–17 years (Group 1) and participants aged 4–11 years (Group 2)

	**Group 1 (Aged 12–17 Years)**		**Group 2 (Aged 4–11 Years)**	
	**Tolvaptan (n=35)**	**Placebo (n=31)**	**Tolvaptan (n=13)**	**Placebo (n=12)**
Age (years)				
Mean (SD)	14.5 (1.7)	14.2 (1.5)	8.7 (2.1)	9.3 (1.7)
Range	12,17	12,17	5,11	6,11
Female, n (%)	18 (51)	16 (52)	3 (23)	7 (58)
Race, n (%)				
White	35 (100)	30 (97)	11 (85)	12 (100)
Black	0	1 (3)	0	0
Asian	0	0	2 (15)	0
Ethnicity, n (%)				
Hispanic or Latino	1 (3)	1 (3)	0	0
Not Hispanic or Latino	34 (97)	30 (97)	13 (100)	12 (100)
Weight (kg), mean (SD)	60.3 (11.0)	57.5 (15.5)	36.4 (14.8)	34.6 (10.6)
Range	32.0,79.0	30.4,108.2	20.7,74.0	23.0,60.1
Height (cm), mean (SD)	168.9 (11.1)	166.5 (10.0)	138.1 (15.3)	139.8 (12.3)
Range	150.0,193.0	141.0,186.0	113.0,166.0	115.0,154.0
Height SDS, mean (SD)	0.47 (1.06)	0.26 (1.02)	0.75 (1.3)	0.39 (0.73)
Body mass index (kg/m^2), mean (SD)	21.1 (3.3)	20.5 (4.2)	18.4 (3.9)	17.4 (3.0)
Range	14.2,28.9	15.3,34.9	14.4,26.9	15.0,26.0
Diagnosis age (years), mean (SD)	6.3 (5.3)	9.4 (4.9)	2.3 (3.6)	3.5 (4.3)
Range	0,17	0,16	0,10	0,11
Genetic testing performed, n (%)	9 (26)	11 (36)	5 (39)	2 (17)
Other blood-related family with PKD, (%)				
Yes	30 (86)	27 (87)	13 (100)	12 (100)
No	4 (11)	4 (13)	0	0
Unknown	1 (3)	0	0	0
Aware of family history before diagnosis, n (%)	31 (89)	27 (87)	12 (92)	12 (100)
Reason for diagnosis, n (%)				
Consequence of ADPKD signs or symptoms	5 (14)	12 (39)	1 (8)	5 (42)
Incidental (due to tests unrelated to ADPKD or its symptoms)	8 (23)	4 (13)	2 (15)	1 (8)
Asymptomatic screening (no prior ADPKD symptoms)	22 (63)	14 (45)	10 (77)	6 (50)
Spot urine osmolality (mOsm/kg), mean (SD)	628 (272)	644 (266)	653 (194)	649 (213)
Urine specific gravity, mean (SD)	1.017 (0.007)	1.016 (0.007)	1.017 (0.004)	1.018 (0.005)
Height-adjusted total kidney volume (mL/cm), n	30	27	9	6
Mean (SD)	3.5 (4.29)	2.7 (0.79)	1.7 (0.72)	2.8 (1.68)
eGFR by bedside Schwartz formula, mL/min/1.73 m^2, mean (SD)	97 (16.8)	101 (13.3)	103 (25.6)	98 (19.3)
ADPKD medical history, n (%)[a]				
Hepatic cysts	4 (11)	2 (7)	0	0
Non–hepato-kidney cysts	1 (3)	0	0	0
Gross hematuria	3 (9)	0	0	0
Upper urinary tract infection	1 (3)	3 (10)	0	0
Proteinuria	6 (17)	6 (19)	1 (8)	1 (8)

Hypertension	6 (17)	10 (32)	1 (8)	4 (33)
Kidney pain	4 (11)	5 (16)	1 (8)	1 (8)

[a]There were no participants with a medical history of nephrolithiasis, anemia, colonic diverticulitis, or vascular/cardiac abnormalities.

ADPKD, autosomal dominant polycystic kidney disease; eGFR, estimated glomerular filtration rate; PKD, polycystic kidney disease; SD , standard deviation; SDS, standard deviation score.

Supplemental Table 3. Modal total daily dose at study visits

	Participants 12–17 Years							
	**Tolvaptan (n=35)**				**Placebo (n=31)**			
**Modal Dose (mg)**	**Week 1 n (%)**	**Month 1 n (%)**	**Month 6 n (%)**	**Month 12 n (%)**	**Week 1 n (%)**	**Month 1 n (%)**	**Month 6 n (%)**	**Month 12 n (%)**
0	0 (0)	0 (0)	0 (0)	0 (0)	0 (0)	1 (3)	0 (0)	0 (0)
7.5	0 (0)	0 (0)	0 (0)	1 (3)	0 (0)	0 (0)	0 (0)	0 (0)
15	0 (0)	0 (0)	0 (0)	1 (3)	0 (0)	0 (0)	0 (0)	0 (0)
22.5	2 (6)	2 (6)	3 (9)	3 (10)	5 (16)	2 (7)	0 (0)	0 (0)
30	1 (3)	0 (0)	0 (0)	0 (0)	0 (0)	0 (0)	0 (0)	0 (0)
37.5	0 (0)	3 (9)	7 (20)	6 (19)	0 (0)	0 (0)	0 (0)	0 (0)
45	30 (86)	15 (43)	13 (37)	12 (39)	22 (71)	10 (32)	13 (42)	12 (40)
60	2 (6)	13 (37)	11 (31)	7 (23)	4 (13)	16 (52)	16 (52)	16 (53)
90	0 (0)	2 (6)	1 (3)	1 (3)	0 (0)	2 (7)	2 (7)	2 (7)
	**Participants <12 Years**							
	**Tolvaptan (n=13)**				**Placebo (n=12)**			
**Modal Dose (mg)**	**Week 1 n (%)**	**Month 1 n (%)**	**Month 6 n (%)**	**Month 12 n (%)**	**Week 1 n (%)**	**Month 1 n (%)**	**Month 6 n (%)**	**Month 12 n (%)**
7.5	0 (0)	1 (8)	1 (8)	1 (8)	0 (0)	0 (0)	0 (0)	0 (0)
15	3 (23)	3 (23)	3 (23)	4 (31)	0 (0)	0 (0)	0 (0)	0 (0)
22.5	7 (54)	5 (39)	5 (39)	4 (31)	10 (83)	4 (33)	4 (33)	3 (25)
30	0 (0)	0 (0)	0 (0)	0 (0)	0 (0)	0 (0)	0 (0)	0 (0)
37.5	0 (0)	0 (0)	1 (8)	1 (8)	0 (0)	0 (0)	0 (0)	0 (0)
45	3 (23)	3 (23)	3 (23)	3 (23)	2 (17)	7 (58)	7 (58)	8 (67)
60	0 (0)	1 (8)	0 (0)	0 (0)	0 (0)	1 (8)	1 (8)	1 (8)
90	0 (0)	0 (0)	0 (0)	0 (0)	0 (0)	0 (0)	0 (0)	0 (0)

Denominators are the number of participants at that specific visit. Modal dose is since the prior visit.

Supplemental Table 4. Criteria for potentially clinically significant changes in laboratory values

		Test Result Grade								
**Parameter**	**Abnormality**	**-4**	**-3**	**-2**	**-1**	**0**	**1**	**2**	**3**	**4**
Creatinine (mg/dL)	Increase					BSL	>BSL–1.5xBSL	>1.5–3xBSL	>3–6xBSL	>6xBSL
Potassium (mEq/L)	Decrease	<2.5	2.5–<3		3–<LLN	LLN				
	Increase					ULN	>ULN–5.5	>5.5–6	>6–7	>7
Sodium (mg/dL)	Decrease	<120	120–124	125–129	130–135	≥136				
	Increase					≤145	146–150	151–155	156–160	>160

Potentially clinically significant increase: BSL grade 0 or 1 and post-BSL grade >1 or BSL grade >1 and post-BSL grade > BSL grade.

Potentially clinically significant decrease: BSL grade 0 or -1 and post-BSL grade <-1 or BSL grade <-1 and post-BSL grade < BSL grade.

BSL, baseline; LLN, lower limit of normal; ULN, upper limit of normal.

Supplemental Table 5. Summary of adverse events and listing of individual treatment-emergent adverse events (by system organ class and MedDRA preferred term) occurring in ≥10% of participants in any treatment arm, participants aged 12–17 years (Group 1) and 4–11 years (Group 2)

	**Aged 12–17 Years**		**Aged <12 Years**	
**n (%)**	**Tolvaptan (n=35)**	**Placebo (n=31)**	**Tolvaptan (n=13)**	**Placebo (n=12)**
Participant days of drug exposure	11788	10766	4714	4294
Participants with AEs	32 (91)	30 (97)	13 (100)	12 (100)
AEs	257	193	87	114
Participants with treatment-emergent AEs	31 (89)	30 (97)	13 (100)	12 (100)
Treatment-emergent AEs[a]	214	154	75	83
Participants with serious treatment-emergent AEs	1 (3)	4 (13)	0	2 (17)
Participants with nonserious treatment-emergent AEs	31 (89)	30 (97)	13 (100)	12 (100)
Participants with severe treatment-emergent AEs	1 (3)	2 (7)	0	0
Participants discontinued study medication due to AEs	1 (3)	1 (3)	0	0
Deaths	0	0	0	0
Gastrointestinal disorders				
Abdominal pain	5 (14)	3 (10)	1 (8)	0
Abdominal pain upper	3 (9)	3 (10)	2 (15)	1 (8)
Constipation	2 (6)	1 (3)	3 (23)	0
Diarrhea	2 (6)	2 (7)	1 (8)	5 (42)
Discolored feces	0	0	2 (15)	0
Nausea	3 (9)	6 (19)	0	1 (8)
Vomiting	4 (11)	6 (19)	3 (23)	4 (33)
General disorders and administration site conditions				
Pyrexia	3 (9)	1 (3)	1 (8)	2 (17)
Thirst	4 (11)	1 (3)	3 (23)	1 (8)
Immune system disorders				
Seasonal allergy	2 (6)	1 (3)	2 (15)	0
Infections and infestations				
Ear infection	1 (3)	4 (13)	0	0
Nasopharyngitis	7 (20)	8 (26)	3 (23)	6 (50)
Pharyngitis	4 (11)	0	0	0
Viral infection	1 (3)	0	2 (15)	1 (8)
Injury, poisoning, and procedural complications				

Skin abrasion	0	0	1 (8)	2 (17)
Investigations				
Serum creatinine increased	8 (23)	0	1 (8)	2 (17)
Serum sodium increased	0	0	2 (15)	0
Vitamin D decreased	0	1 (3)	2 (15)	0
Metabolism and nutrition disorders				
Polydipsia	4 (11)	1 (3)	1 (8)	0
Musculoskeletal and connective tissue disorders				
Back pain	4 (11)	5 (16)	0	0
Pain in extremity	0	5 (16)	0	1 (8)
Nervous system disorders				
Dizziness	3 (9)	4 (13)	0	1 (8)
Headache	12 (34)	15 (48)	4 (31)	6 (50)
Kidney and urinary disorders				
Nocturia	4 (11)	2 (7)	3 (23)	1 (8)
Pollakiuria	7 (20)	0	2 (15)	0
Polyuria	11 (31)	1 (3)	2 (15)	1 (8)
Respiratory, thoracic, and mediastinal disorders				
Cough	3 (9)	2 (7)	4 (31)	3 (25)
Oropharyngeal pain	2 (6)	2 (7)	2 (15)	4 (33)
Rhinorrhea	2 (6)	0	0	2 (17)
Skin and subcutaneous tissue disorders				
Rash	0	0	0	2 (17)
Vascular disorders				
Hypertension	4 (11)	1 (3)	0	0
Orthostatic hypotension	5 (14)	0	0	0

Individual AEs occurred in ≥10% of participants in any treatment group in either age group.

[a]Defined as an AE that started after the initiation of study medication; or if the event was continuous from baseline and was serious, study medication-related, or resulted in death, discontinuation, interruption, or reduction of study medication. Multiple occurrences of treatment-emergent AEs are counted once per MedDRA preferred term.

AE, adverse event; MedDRA, Medical Dictionary for Regulatory Activities.

Supplemental Table 6. Summary of adverse events and listing of individual treatment-emergent adverse events (by system organ class and MedDRA preferred term) occurring in ≥5% of tolvaptan-treated participants, full safety population and excluding participants with verified nonadherence

**n (%)**	**Tolvaptan Safety Set (n=48)**	**Tolvaptan-Adherent Only (n=42)**
Participant days of drug exposure	16502	14212
Participants with AEs	45 (94)	39 (93)
AEs	344	276
Participants with treatment-emergent AEs	44 (92)	38 (91)
Treatment-emergent AEs[a]	289	236
Participants with serious treatment-emergent AEs	1 (2)	1 (2)
Participants with nonserious treatment-emergent AEs	44 (92)	38 (91)
Participants with severe treatment-emergent AEs	1 (2)	1 (2)
Participants discontinued study medication due to AEs	1 (2)	1 (2)
Deaths	0	0
Ear and labyrinth disorders		
Ear pain	3 (6)	2 (5)
Gastrointestinal disorders		
Abdominal pain	6 (13)	4 (10)
Abdominal pain upper	5 (10)	4 (10)
Constipation	5 (10)	5 (12)
Diarrhea	3 (6)	2 (5)
Nausea	3 (6)	1 (2)
Vomiting	7 (15)	6 (14)
General disorders and administration site conditions		
Fatigue	4 (8)	3 (7)
Pyrexia	4 (8)	4 (10)
Thirst	7 (15)	7 (17)
Immune system disorders		
Seasonal allergy	4 (8)	4 (10)
Infections and infestations		
Bronchitis	3 (6)	2 (5)
Nasopharyngitis	10 (21)	8 (19)
Pharyngitis	4 (8)	2 (5)
Rhinitis	3 (6)	2 (5)
Upper respiratory tract infection	4 (8)	3 (7)
Viral infection	3 (6)	3 (7)
Investigations		
Serum creatinine increased	9 (19)	8 (19)
Metabolism and nutrition disorders		
Decreased appetite	4 (8)	4 (10)
Polydipsia	5 (10)	4 (10)
Musculoskeletal and connective tissue disorders		
Back pain	4 (8)	2 (5)
Nervous system disorders		
Dizziness	3 (6)	1 (2)
Headache	16 (33)	11 (26)
Kidney and urinary disorders		

Nocturia	7 (15)	7 (17)
Pollakiuria	9 (19)	7 (17)
Polyuria	13 (27)	12 (29)
Respiratory, thoracic, and mediastinal disorders		
Cough	7 (15)	7 (17)
Epistaxis	3 (6)	2 (5)
Oropharyngeal pain	4 (8)	3 (7)
Vascular disorders		
Hypertension	4 (8)	3 (7)
Orthostatic hypotension	5 (10)	5 (12)

Individual AEs shown occurred in ≥5% of tolvaptan-treated participants in the full safety population. No additional individual AEs occurred in ≥5% of the tolvaptan-adherent subgroup.

[a]Defined as an AE that started after the initiation of study medication; or if the event was continuous from baseline and was serious, study medication-related, or resulted in death, discontinuation, interruption, or reduction of study medication. Multiple occurrences of treatment-emergent AEs are counted once per MedDRA preferred term.

AE, adverse event; MedDRA, Medical Dictionary for Regulatory Activities.

Supplemental Table 7. Mean change from baseline in height standard deviation scores (SDS) by age and sex (reference: Northern European populations)

	Tolvaptan		Placebo	
**Group and Visit[a]**	**n[b]**	**Mean (SD)**	**n[b]**	**Mean (SD)**
Overall population				
Baseline growth percentile	48	0.55 (1.12)	43	0.30 (0.94)
Change from Baseline to Month 12	44	0.54 (0.56)	40	0.59 (0.61)
Females aged 15-17 years				
Baseline growth percentile	9	0.49 (1.36)	8	-0.05 (1.11)
Change from Baseline to Month 12	9	0.07 (0.11)	7	0.07 (0.12)
Males aged 15-17 years				
Baseline growth percentile	9	0.28 (1.15)	8	0.08 (0.64)
Change from Baseline to Month 12	8	0.22 (0.25)	8	0.37 (0.49)
Females aged 12-14 years				
Baseline growth percentile	9	0.38 (0.74)	8	0.12 (1.27)
Change from Baseline to Month 12	7	0.15 (0.23)	8	0.58 (0.41)
Males aged 12-14 years				
Baseline growth percentile	8	0.77 (1.05)	7	0.97 (0.81)
Change from Baseline to Month 12	7	1.06 (0.76)	5	0.39 (1.06)
Females aged 4-11 years				
Baseline growth percentile	3	1.18 (1.00)	7	0.41 (0.64)
Change from Baseline to Month 12	3	1.09 (0.29)	7	1.13 (0.30)
Males aged 4-11 years				
Baseline growth percentile	10	0.62 (1.40)	5	0.37 (0.92)
Change from Baseline to Month 12	10	0.98 (0.24)	5	1.11 (0.41)

[a]Baseline is the last available predose evaluation.
[b]For the baseline visit, n is the total number of treated participants with a predose evaluation. For the Month 12 visit, n is the total number of treated subjects with both baseline and Month 12 evaluations.
Reference values are from: Bonthuis M et al. Use of national and international growth charts for studying height in European children: development of up-to-date European height-for-age charts. *PLoS One*. 2012;7:e42506.

Supplemental Table 8. Tanner staging at baseline and Month 12 in participants aged 12–17 years (Group 1) and 4–11 years (Group 2)

n (%)	Classification	Group 1 (Aged 12–17 Years) Tolvaptan (n=35)	Group 1 (Aged 12–17 Years) Placebo (n=31)	Group 2 (Aged 4–11 Years) Tolvaptan (n=13)	Group 2 (Aged 4–11 Years) Placebo (n=12)
Baseline	Stage 1	1 (2.9)	0 (0.0)	11 (84.6)	7 (58.3)
	Stage 2	3 (8.6)	4 (12.9)	0 (0.0)	2 (16.7)
	Stage 3	3 (8.6)	3 (9.7)	1 (7.7)	1 (8.3)
	Stage 4	5 (14.3)	4 (12.9)	0 (0.0)	0 (0.0)
	Stage 5	20 (57.1)	18 (58.1)	0 (0.0)	0 (0.0)
Month 12	Stage 1	0 (0.0)	0 (0.0)	9 (69.2)	5 (41.7)
	Stage 2	0 (0.0)	2 (6.5)	2 (15.4)	2 (16.7)
	Stage 3	1 (2.9)	1 (3.2)	0 (0.0)	2 (16.7)
	Stage 4	6 (17.1)	5 (16.1)	1 (7.7)	1 (8.3)
	Stage 5	23 (65.7)	20 (64.5)	0 (0.0)	0 (0.0)

Baseline is the last pre-dose evaluation.

Supplemental Table 9. Scores on the Pediatric Quality of Life Inventory (PedsQL™) Acute Version 4.0 Generic Core Scale and PedsQL™ Multidimensional Fatigue Scale Acute Version 3.0

Outcome	All Participants				Participants Aged 12–17 years*			
	Tolvaptan		Placebo		Tolvaptan		Placebo	
	n	Mean Score (SD)	n	Mean Score (SD)	n	Mean Score (SD)	n	Mean Score (SD)
Generic Core Scale								
Baseline	35	84.3 (9.9)	33	86.2 (10.7)	35	84.3 (9.9)	31	85.8 (10.9)
Month 12	28	90.0 (9.0)	26	87.8 (12.4)	28	90.0 (9.0)	26	87.8 (12.4)
Multidimensional Fatigue Scale								
Baseline	34	77.9 (15.1)	33	74.0 (15.9)	34	77.9 (15.1)	31	73.9 (16.4)
Month 12	29	83.2 (15.0)	28	81.5 (18.4)	29	83.2 (15.0)	28	81.5 (18.4)

*Data on participants aged 4–11 years not shown. There were only 2 participants aged 4–11 (placebo) with PedsQL data.

Supplemental Figure 1. Participant disposition by age group

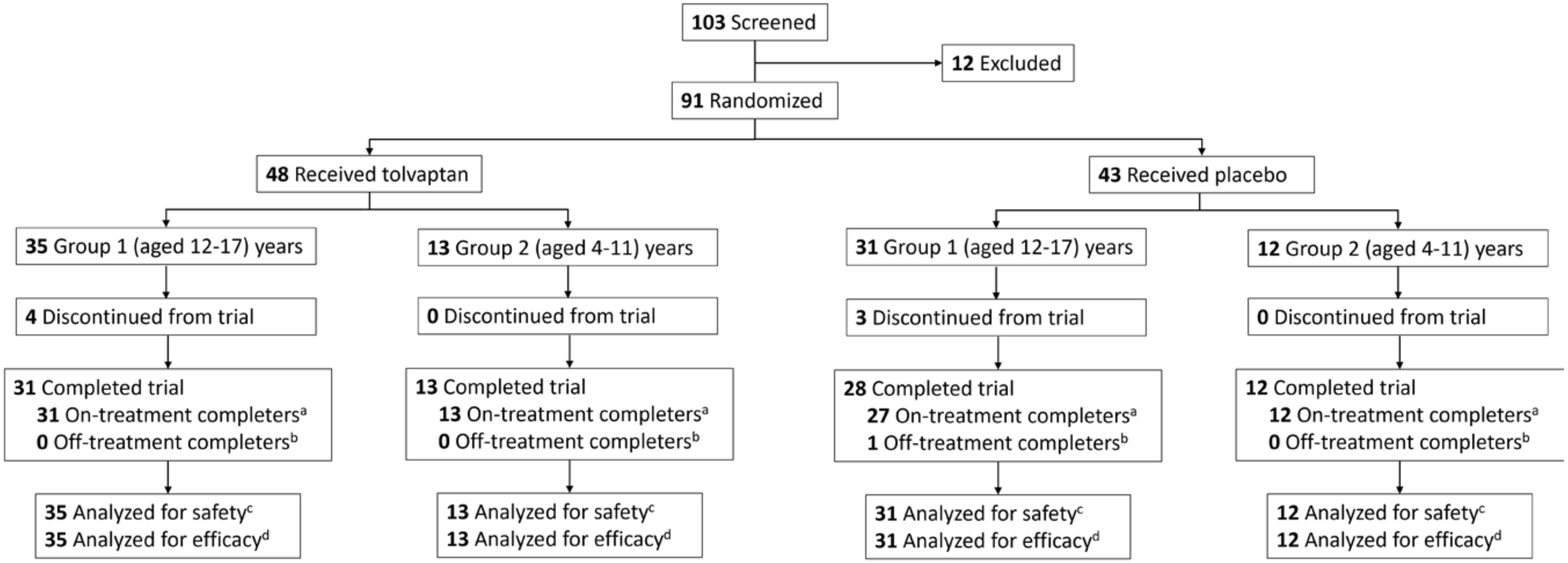

[a]Randomized participants who completed the Month 12 visit on study medication. [b]Randomized participants who discontinued study medication prior to the Month 12 visit but continued with trial visit assessments. [c]All participants who were randomized and took ≥1 dose of study medication after randomization. [d]All participants who were in the randomized population, took ≥1 dose of study medication after randomization, and had a baseline and ≥1 valid post-baseline efficacy evaluation.

Supplemental Figure 2. Change from baseline in spot urine osmolality (mOsm/kg) and urine specific gravity in (A) participants aged 12–17 years (Group 1) and (B) participants aged 4–11 years (Group 2)

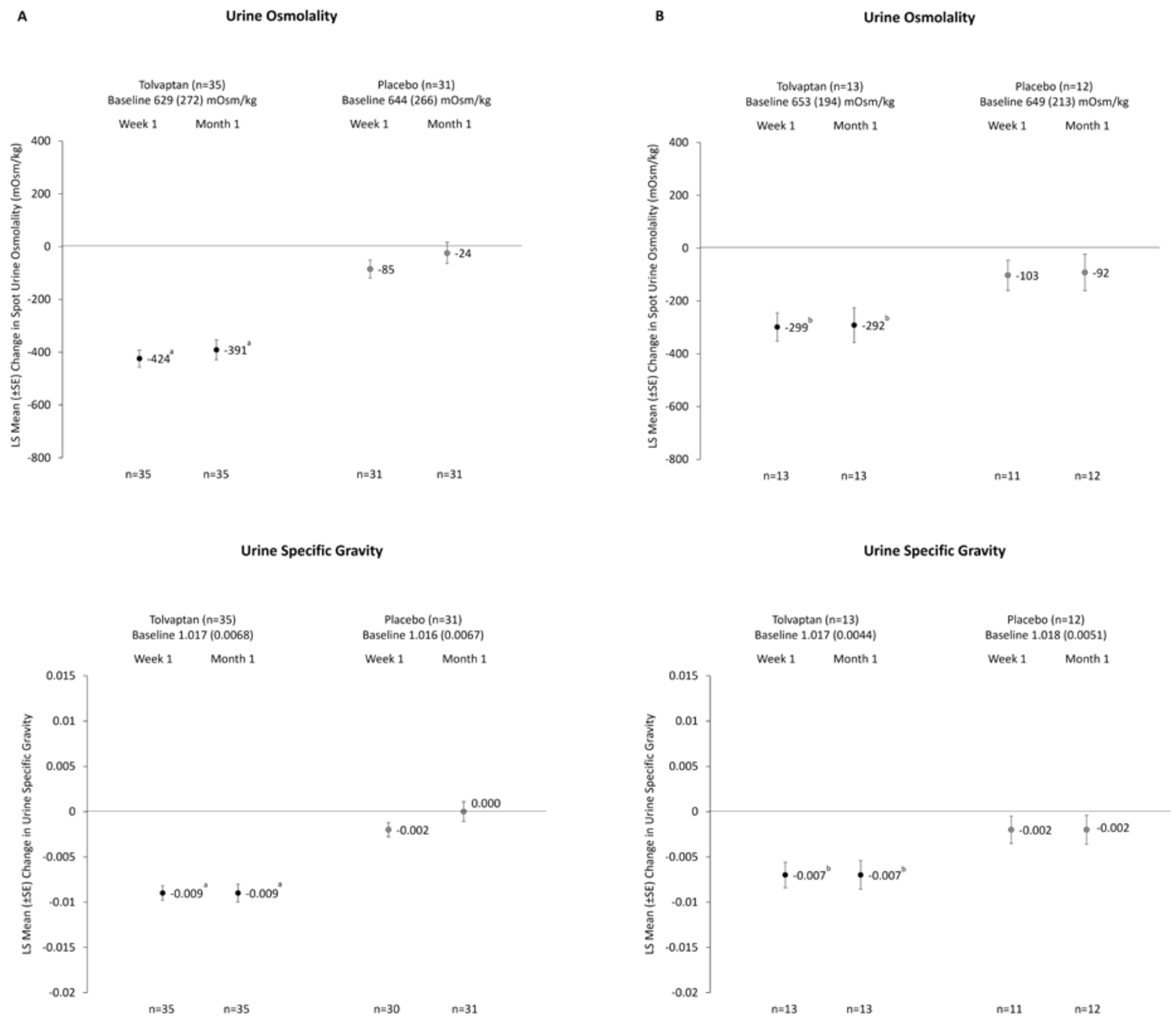

Baseline value is mean (± standard deviation). [a]$P<0.001$, [b]$P<0.05$ for tolvaptan vs placebo.

LS, least squares; SE, standard error.

Supplemental Figure 3. Percent change from baseline to Month 12 in htTKV on MRI (in participants aged 12–17 years) and ultrasound (in participants aged 4–11 years), all participants

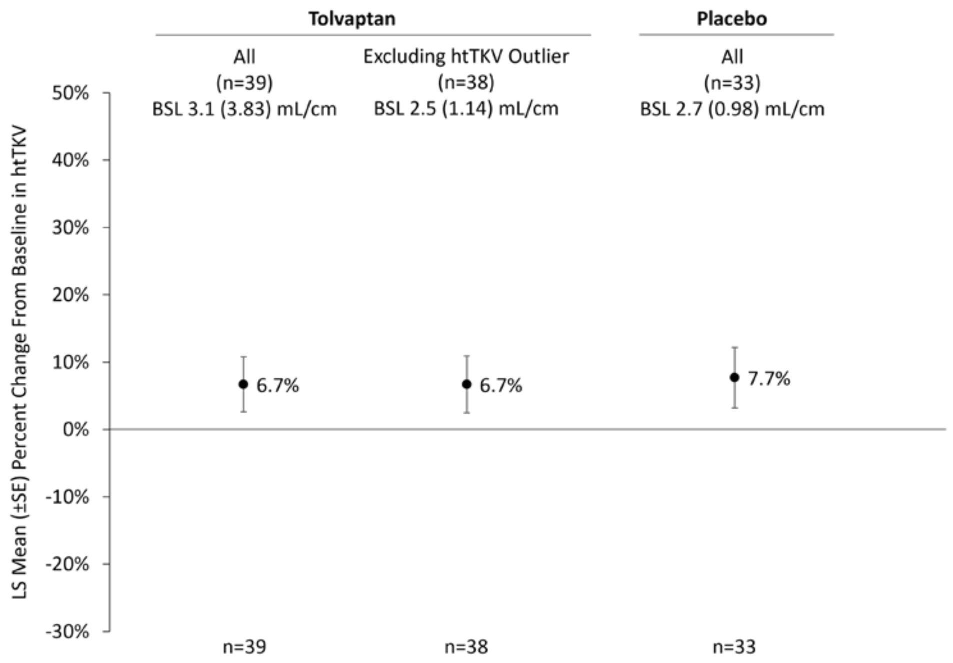

Baseline value is mean (± standard deviation).

For each tolvaptan group vs placebo, *P*>0.05.

BSL, baseline; htTKV, height-adjusted total kidney volume; LS, least squares; MRI, magnetic resonance imaging; SE, standard error.

Supplemental Figure 4. Percent change from baseline to Month 12 in htTKV on ultrasound in participants aged 4–11 years (Group 2)

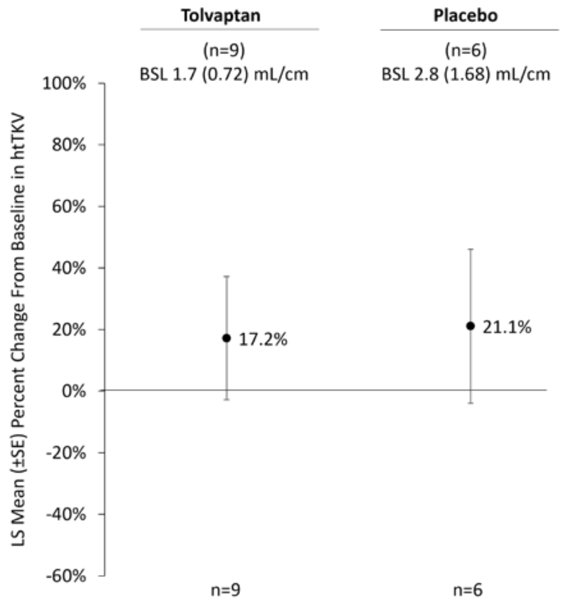

Baseline value is mean (± standard deviation).

For tolvaptan vs placebo, *P*=0.91.

BSL, baseline; htTKV, height-adjusted total kidney volume; LS, least squares; SE, standard error.

Supplemental Figure 5. Change in eGFR from Week 1 through Month 12, all participants excluding tolvaptan participants with verified nonadherence

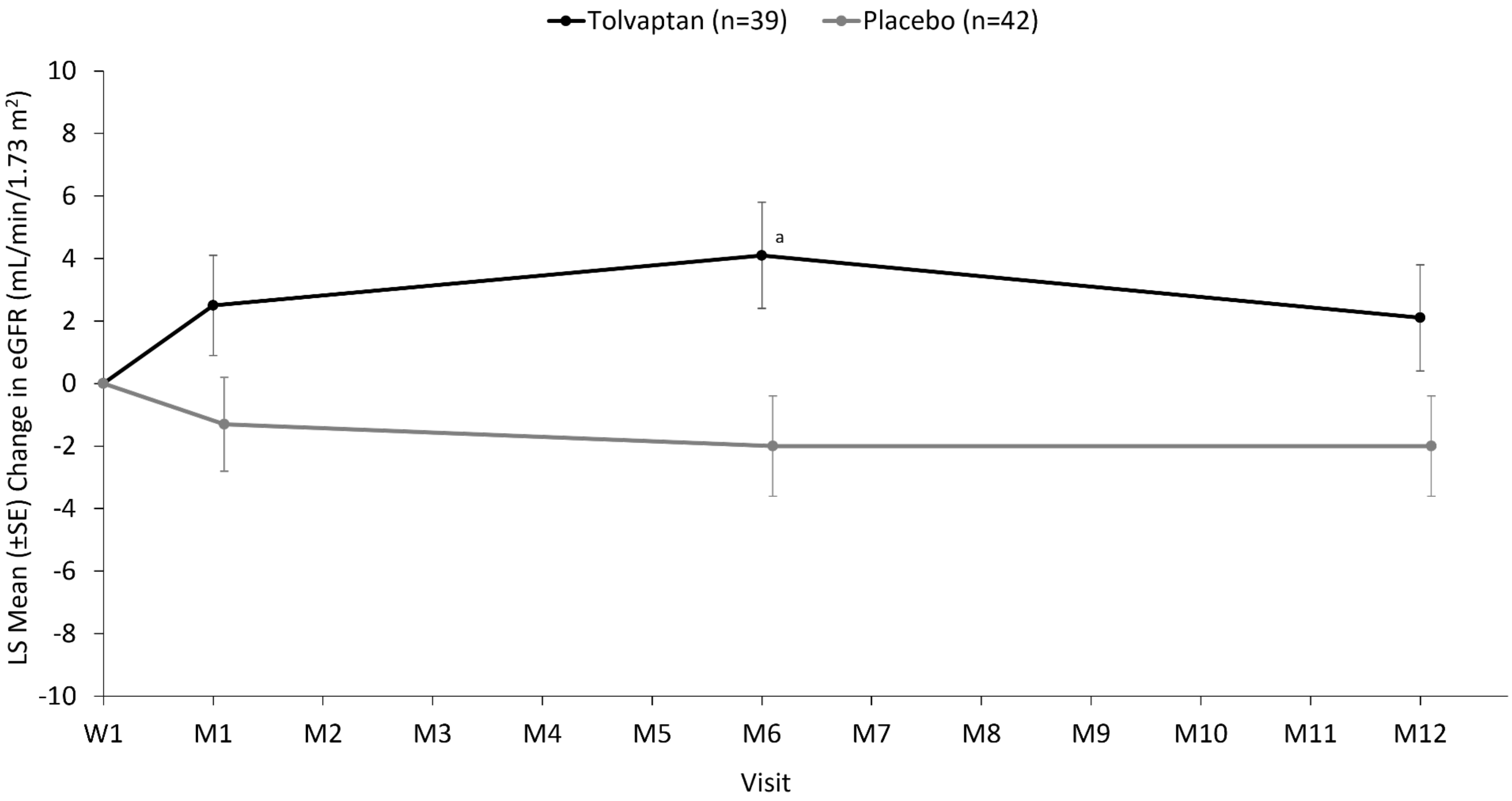

[a]P<0.05 for tolvaptan vs placebo.

eGFR, estimated glomerular filtration rate; LS, least squares; M, month; SE, standard error; W, week.

Supplemental Figure 6. Change in eGFR from Week 1 through Month 12, participants aged 12–17 years (Group 1)

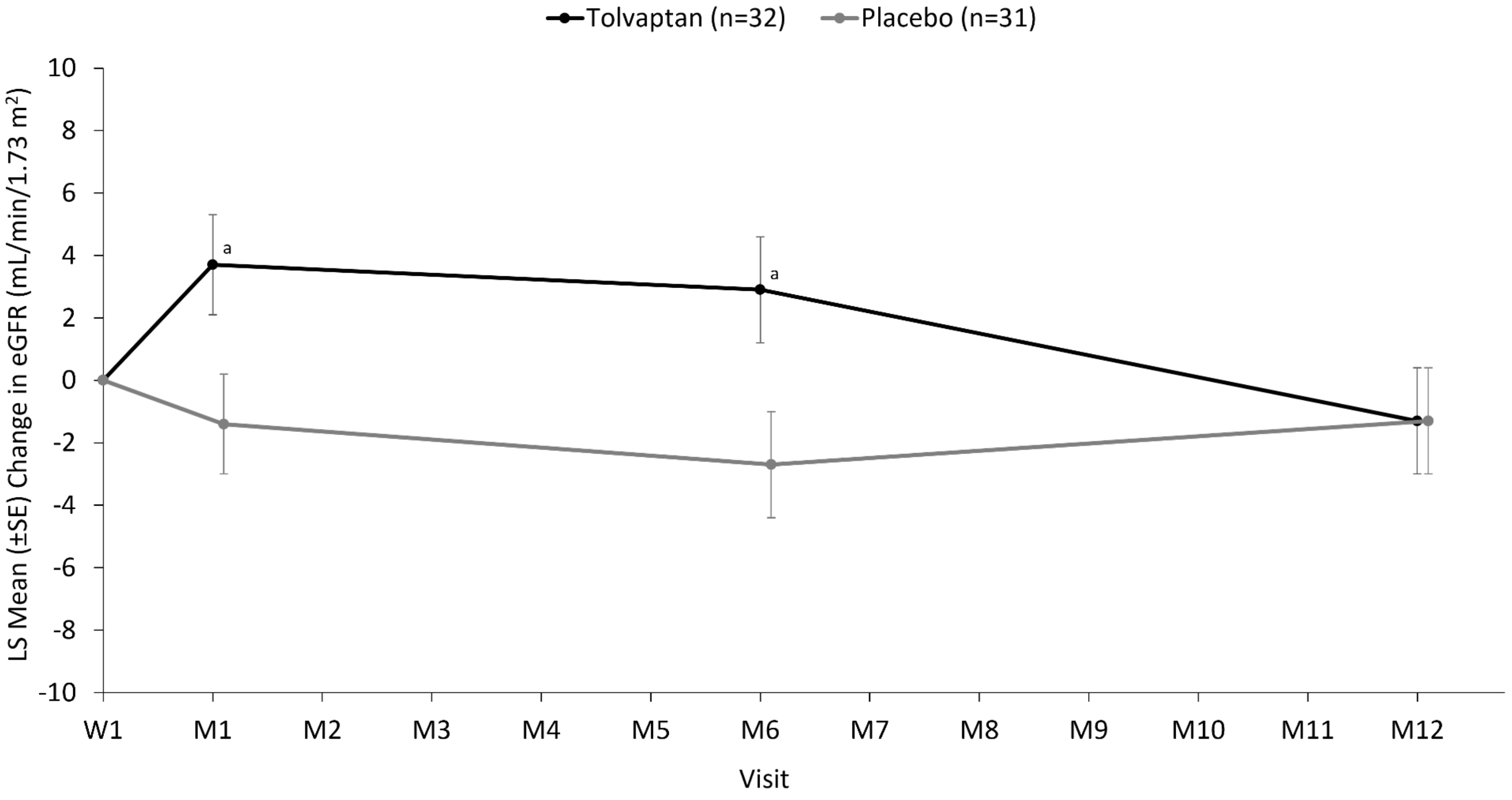

[a]*P*<0.05 for tolvaptan vs placebo.

eGFR, estimated glomerular filtration rate; LS, least squares; M, month; SE, standard error; W, week.

Supplemental Figure 7. Change in eGFR from Week 1 through Month 12, participants aged 4–11 years (Group 2)

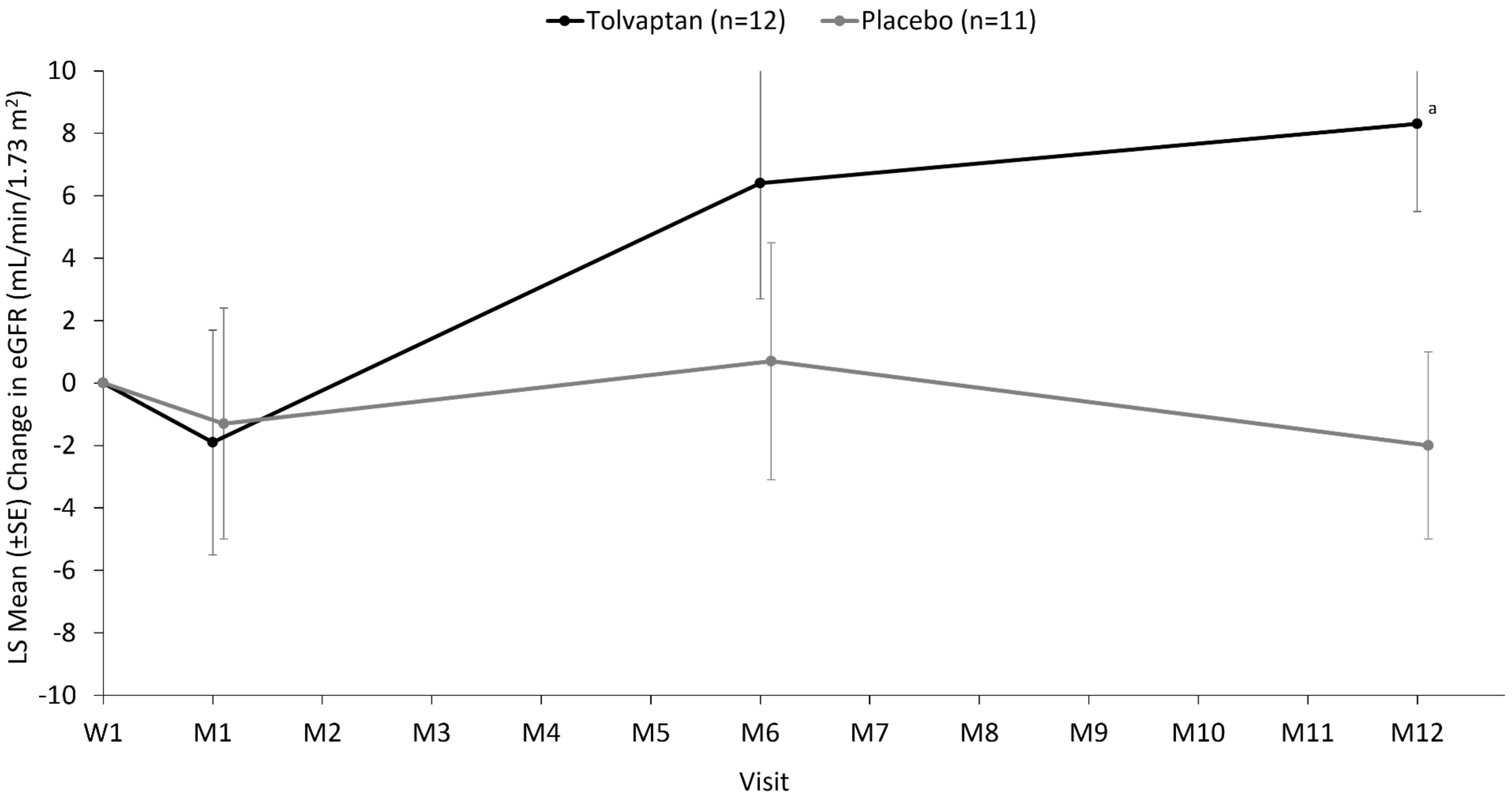

[a]$P<0.05$ for tolvaptan vs placebo.

eGFR, estimated glomerular filtration rate; LS, least squares; M, month; SE, standard error; W, week.

Supplemental Figure 8. Mean daytime and nighttime number of voids from in participants aged 12–17 years (Group 1)

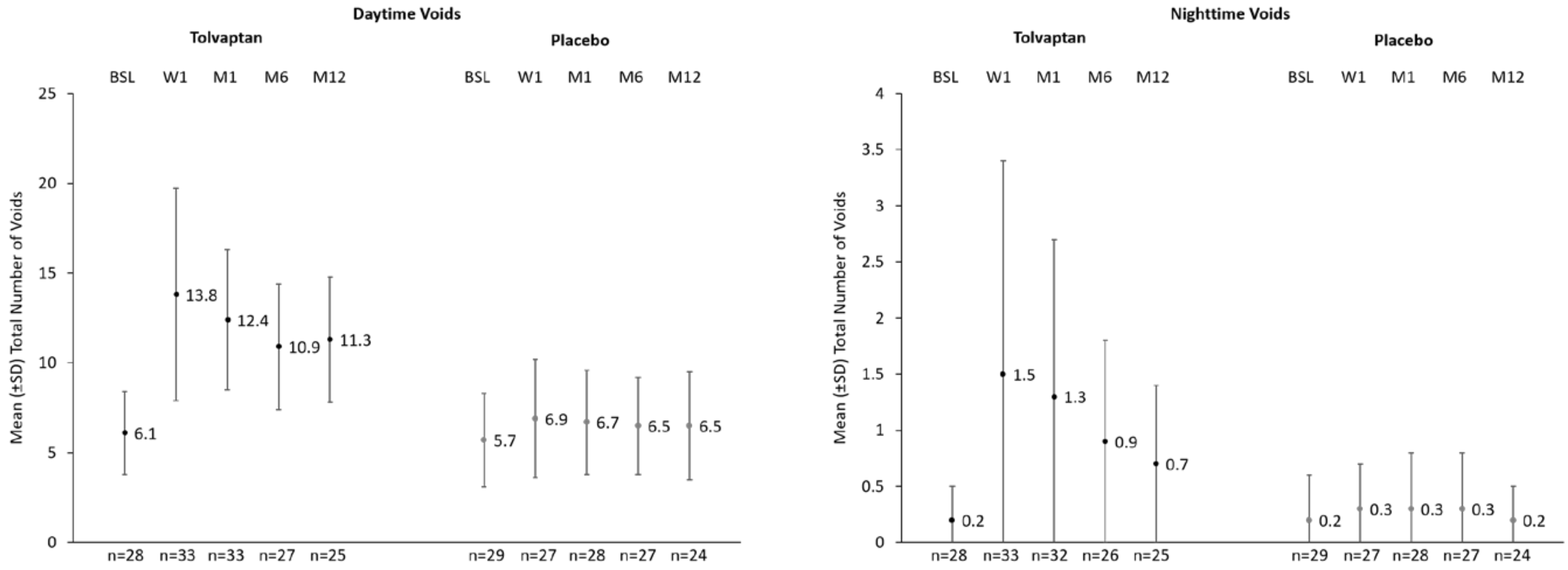

Voids data on participants aged 4–11 years (Group 2) were limited (1–3 participants in each treatment arm) and are not shown.

BSL, baseline; M, month; W, week.
